# Antioxidant capacity and cytotoxic effect of an optimized extract of isabella grape (*Vitis labrusca*) on breast cancer cells

**DOI:** 10.1016/j.heliyon.2023.e16540

**Published:** 2023-05-24

**Authors:** M. Daniela Vélez, María A. Llano-Ramirez, Carolina Ramón, Jessica Rojas, Carolina Bedoya, Sandra Arango-Varela, Gloria A. Santa-González, Maritza Gil

**Affiliations:** a Grupo de Investigación e Innovación Biomédica, Facultad de Ciencias Exactas y Aplicadas, Instituto Tecnológico Metropolitano, Medellín 050034, Colombia; b Química Básica, Aplicada y Ambiente Alquimia, Facultad de Ciencias Exactas y Aplicadas, Instituto Tecnológico Metropolitano, Medellín 050034, Colombia; c Didáctica y Modelamiento en Ciencias Exactas y Aplicadas (DAVINCI), Facultad de Ciencias Exactas y Aplicadas, Instituto Tecnológico Metropolitano, Medellín 050034, Colombia; d Food Engineering Research Group, Unilasallista Corporación Universitaria, Caldas 055440, Colombia

**Keywords:** *Vitis labrusca*, Phenolic compounds, Ultrasound, Breast cancer, Apoptosis, Molecular docking

## Abstract

The phenolic profile of Isabella grape (*Vitis labrusca*) offers beneficial properties to human health and makes it a functional food product. In order to better understand the phenolic compounds found in this grape variety and the biological effect they induce on breast cancer cells, an ultrasound-assisted extraction was carried out. During the extraction of polyphenols from Isabella grapes organically grown in Antioquia (Colombia), parameters such as frequency (33 kHz and 40 kHz), time and solvent were optimized to finally obtain a crude extract with antioxidant properties (Oxygen Radical Absorbance Capacity, ORAC: 293.22 ± 34.73 μmol of Trolox/g of sample), associated with a total polyphenol content (TPC) of 43.14 ± 5.00 mg GAE/g sample and a total anthocyanin content composed of 17.69 ± 2.59 mg of malvidin-3-glucoside/100 g of sample. MCF-7 breast cancer cells were treated with different concentrations of the optimized extract, and results show a decrease in cell viability related to mitochondrial membrane depolarization, ROS increase, and chromatin condensation. To determine the possible death induction mechanism, molecular docking was simulated to predict the molecular interactions between the most abundant phenolic compounds in Isabella grape and the main apoptosis-related proteins. The results obtained from *in silico* and *in vitro* experiments were consistent with each other, suggesting that the phenolic compounds found in Isabella grape can be considered potential adjuvant chemopreventive agents for the treatment of breast cancer.

## 1 Introduction

Functional food products are those that, besides offering nutritional value, have at least one benefit to human health [1,2]. Multiple studies have explored fruits and vegetables that could be considered functional foods thanks to their phytochemical content [3]. Specifically, Isabella grape (*V. labrusca*) has high contents of phenolic compounds, such as anthocyanins and flavonoids, whose active centers can capture unpaired electrons, which explains its great antioxidant potential [[4], [5], [6], [7]]. Since the properties of phytochemicals were discovered, efficient methodologies have been sought to extract and isolate them without altering their potential antioxidant effect, making it possible to transform their active compounds into additives for different uses. According to the literature, ultrasound-assisted extraction is one of the most efficient methods for obtaining phenolic compounds, thanks to the short times it requires to achieve a high recovery yield compared to methods such as soxhlet and solid-liquid, which require up to 24 h [[8], [9], [10]]. In addition, this method avoids the use of toxic organic solvents, which cannot be employed in the pharmaceutical, cosmetic, or food industries, in accordance with the regulations established in the Codex Alimentarius [11].

Due to the biological protective effect that functional foods have on cancer development, Isabella grape has been studied as a potential chemopreventive and therapeutic agent for the treatment of different neoplasms. The chemopreventive mechanisms reported for this grape variety include cytotoxicity, cell cycle modulation, prooxidant action, apoptosis induction, and regulation of hormone receptors [[12], [13], [14], [15], [16], [17], [18]]. At this point, it is worth mentioning that breast cancer is the most frequently diagnosed type of neoplasm worldwide [19]. The development of this disease is associated with factors such as age, menstruation, menopause, lifestyle (poor diet, smoking, physical inactivity), family history and genetic alterations [20,21].

Apoptosis is a defense mechanism that eliminates mutated, damaged, and/or harmful cells that precede carcinogenesis; it occurs in an orderly and controlled manner thanks to a complex network of antiapoptotic and proapoptotic proteins and effector molecules that are involved throughout the process [22,23]. Certain phenolic compounds have been reported to induce changes in these proteins' expression, thus activating the different apoptosis pathways [24,25]. Therefore, to explore the interaction between these compounds and apoptosis-related proteins, this study implemented molecular docking, which can reveal and predict the way certain substances interact with ligands of interest, favoring the optimization of resources and time [[26], [27], [28]].

This study aimed to evaluate the cytotoxic and antitumor activity of phenolic compounds extracted from Isabella grapes by *in vitro* analysis and *in silico* prediction, in order to study the functional properties of the berry bioactive compounds on breast cancer cells. Three main stages were proposed and developed throughout the article. The first of these was the optimization of the operational parameters of the ultrasound-assisted extraction of phenolic compounds present in Isabella grape. The second was to evaluate the biological effect of the extract on MCF-7 cells derived from breast adenocarcinoma. Finally, the molecular interactions between the main phenolic compounds found in said grape and the apoptosis-related proteins were examined using *in silico* prediction.

## 2 Materials and methods

### 2.1 Extraction

#### 2.1.1 Materials

The analytical grade reagents used were Merck brand and consisted of: ethanol (CAS N° 64-17-5), acetic acid (CAS N° 64-19-7), methanol (CAS N°67-56-1), hydrochloric acid (CAS N° 7647-01-0), sodium carbonate (CAS N°497-19-8), gallic acid (CAS N° 149-91-7), Folin–Ciocalteu reagent, and fluorescein (CAS N°2321-07-5). The reagents used were Sigma-Aldrich brand and consisted of: Trolox (CAS N° 238-81-3) and AAPH or 2,2′-azobis (2 -amidinopropane) dihydrochloride (CAS N°CAS No.:2997-92-4).

#### 2.1.2 Sample preparation

The organic Isabella grapes (*V. labrusca*), which were free of agrochemicals such as pesticides and cultivated at 1579 m above sea level in Medellín (Antioquia, Colombia), were purchased from Habitat Verde. The grapes were washed with abundant water, disinfected with a 5-mg/L hypochlorite solution, separated from the stem, dried in a Memmert oven (model UN55, Germany), and left at 40 °C until reaching a moisture of 6% ± 1. The dehydrated grapes were then homogenized in a KitchenAid grinder (model BCG111OB, 160 V) at maximum speed. Finally, they were sieved to select the material reduced to a particle size of 250 μm.

#### 2.1.3 Bromatological analysis

The grapes (fresh and dried) were characterized by proximal analysis based on the official standard methods reported by the Association of Analytical Communities, AOAC: (AOAC 972.15: ash), (AOAC 930.20: crude fiber), (AOAC 925.07: ethereal extract) and total nitrogen and protein content (AOAC 970.22: ethereal extract).

#### 2.1.4 Optimization of extraction parameters

The main aim of the first stage of this research work was to optimize the process parameters to maximize the extraction of the total content of polyphenols present in the dried and sieved Isabella grapes by ultrasound-assisted extraction (USAE). Firstly, the extraction solvent was evaluated and, after this parameter was chosen, the extraction time was optimized by simultaneously evaluating two acoustic operating frequencies. The methods carried out for the optimization of the afore-mentioned parameters are described below.

##### 2.1.4.1 Solvent selection for the extraction of total polyphenol content from dried isabella grape by USAE

The selection of the solvent used in the extraction of the total polyphenol content present in previously dried and sieved Isabella grapes was carried out by means of extraction in an ultrasonic bath (GT Sonic, model GT-173QS). Two solvents (ethanol and water) were evaluated, individually and in mixture (Table 1). Each solvent treatment was also evaluated at two acoustic frequencies (33 and 40 kHz). The selection of the solvent type was based on the highest total polyphenols content obtained.

**Table 1:** Evaluation conditions for the selection of solvent for the extraction of total polyphenol content from dried and sieved Isabella grapes.

Treatment^a^	Solvent	Solvent ratio	Frequency (kHz)
1	Water: Ethanol	1:0	33
2	Water: Ethanol	0:1	33
3	Water: Ethanol	0.5:0.5	33
4	Water: Ethanol	1:0	40
5	Water: Ethanol	0:1	40
6	Water: Ethanol	0.5:0.5	40

a Each treatment was carried out in triplicate. (6 treatment x 3 replicates, 18 experiments).

Each evaluated treatment shown in Table 1 was carried out, keeping the following parameters constant throughout the ultrasound-assisted extraction: operating temperature (24 °C ± 1), sonication power (100 W), process time (30 min), and liquid to solid ratio (5:1, or 1.5 mL of solvent to 0.3 g of dried grape). At the end of each USAE run, the as-obtained mixture was centrifuged for 5 min at 4032×*g*, and the supernatant was recovered and stored at 4 °C as suggested by Drosou et al. (2015) [29]. Each treatment was carried out in triplicate. (6 treatment x 3 replicates, 18 experiments).

##### 2.1.4.2 Selection of extraction time and the acoustic frequency

The optimization of the time and frequency by USAE of the total polyphenol content of dried grapes was carried out following a methodology proposed by González-Centeno et al. (2014) [29], with some modifications. Briefly, the extraction was carried out for 15, 30, 45, 60, 75, 90, 105, 120, 135 min, and each extraction time was evaluated at frequencies of 33 and 40 kHz in the ultrasonic bath. All the other parameters of ultrasonic assisted extraction were set as per the study of solvent selection. The optimum extraction time and frequency were determined according to the maximum total polyphenol content. Each treatment was carried out in quadruplicate (18 treatment x 4 replicates, 72 experiments).

#### 2.1.5 Extraction of TPC from isabella grapes under optimized operating parameters

**Fig. 1:**
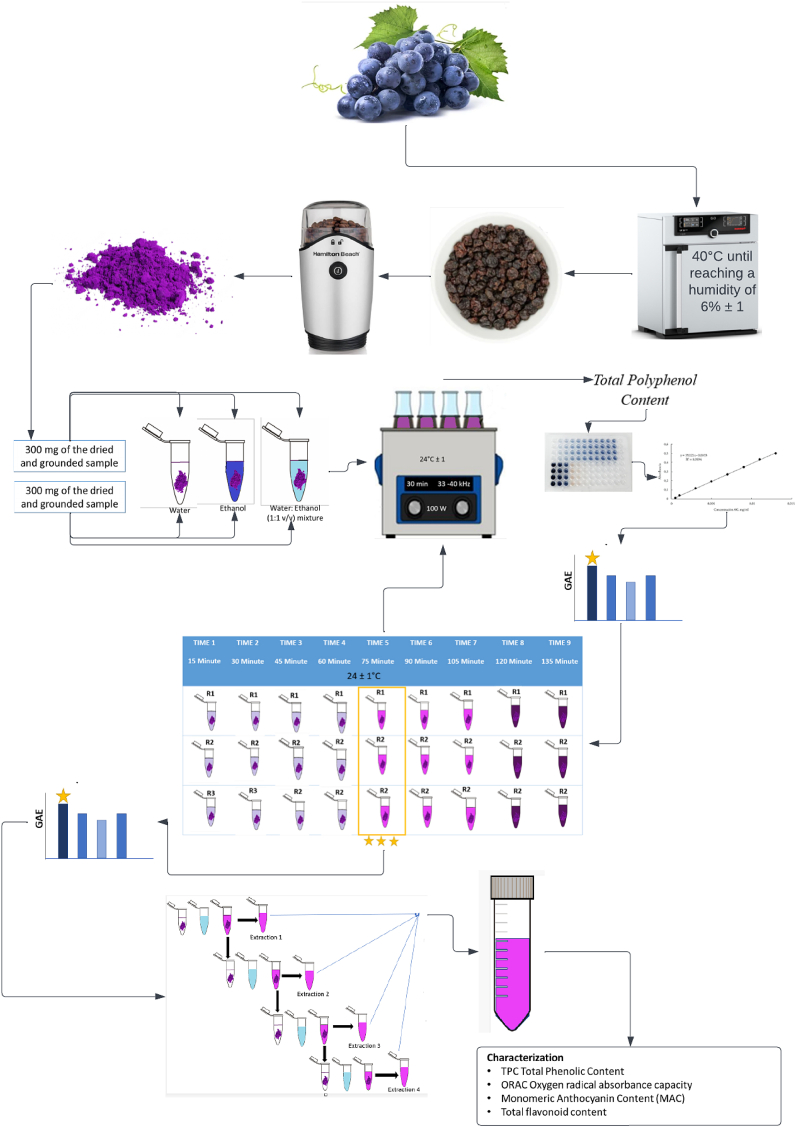
Schematic illustration of ultrasound-assisted extraction (USAE) process to obtain total polyphenols from Isabella grapes under optimized operating parameters (type of solvent, frequency, and time of extraction).

The extraction was carried out under the conditions selected as optimal according to the procedure described in section 2.1.4. The supernatant obtained at the end of the first extraction cycle was separated from the solid plant tissue and reserved. Then, another cycle was started, adding the same amount of solvent (1.5 mL) on the residual plant tissue, and repeating the USAE on this mixture to obtain the second fraction of extract. A third extraction cycle was performed on the same tissue until the solvent became colorless, after which the supernatants were combined in a total extract and used for determination of total phenolic compounds, total flavonoid compounds, monomeric anthocyanins content and ORAC oxygen radical absorbance capacity. This method was adopted from the re-extraction performed by Refs. [30,31] with some modifications. Each experimental run was performed in triplicate. Fig. 1 shows the schematic illustration of the ultrasound-assisted extraction (USAE) process.

#### 2.1.6 Chemical and functional characterization of isabella grape extract

##### 2.1.6.1 Total polyphenol content

This method was carried out to determine the content of total polyphenols in each of the optimization stages (*sections 2.14 and 2.1.4.2*) and in the evaluation of the final extract (under optimal conditions and subjected to exhaustion - item 2.1.5). The TPC was measured using the Folin–Ciocalteu method described by Singleton and Rossi (1965) [32]. A mixture was prepared of 30 μL of gallic acid (10, 20, 40, 60, 80, and 100 μg/mL) and 30 μL of the sample (1:100 sample: distilled water, respectively). The mixture was loaded on a 96-well plate. Subsequently, 30 μl of 10% sodium carbonate (w/v) and 15 μl of Folin–Ciocalteu reagent were added, except in the case of the blank, which consisted of water and sodium carbonate. The mixture was then incubated in the dark at 25 °C for 60 min, and the absorbance was recorded at 760 nm on the plate reader of a spectrofluorometer (Sinergy HT, US). The TPC was expressed as milligrams of gallic acid equivalents per gram of sample (mg GAE/g sample). All measurements were taken in triplicate. The calibration curve was constructed using a linear regression equation. The interpolated values represent the concentration of μg GAE/mL sample. The calculation model to express the results as GAE (mg/g) is shown in Eq. (1).

(1) GAEmggdry=(μgmLofthecurve)(1mg1000μg)(DF)gsample

The GAE values of the three independent experiments are expressed as mean ± standard deviation.

##### 2.1.6.2 Antioxidant capacity

The method implemented here to determine antioxidant capacity was based on previous reports by Ou et al. (2001) with some adjustments [33]. The standard for the construction of the calibration curve was Trolox at concentrations of 10, 25, 50, 100, 150, 200, 300, 400, and 500 μM in a 10-mM phosphate buffer (pH 7.4). Fluorescein, phosphate buffer, the respective dilution of Trolox, and the sample previously diluted in a 1:100 ratio using distilled water were added to a 96-well microplate in that strict order. The mixture was transferred to the plate reader, where it was incubated at 37 °C for 30 min. After this time, the AAPH solution prepared during the incubation time was added to each well. The fluorescence intensity was measured in triplicate every 2 min for 2 h at an excitation wavelength of 485 nm (aperture of 5), an emission wavelength of 520 nm (aperture of 13), and with a 1% attenuator.

The protective effect of the antioxidant was measured based on the fluorescence area under the curve (AUC) of the sample compared to that of the blank, in which no antioxidant was present. The AUC is calculated using Eq. (2).

(2) $$AUC=\left(0.5+\left(\sum_{i=1}^{i=31}\frac{f_{i}}{f_{1}}\right)\right)x\;CT$$

I: number of cycles, F: fluorescence units, CT: cycle time in min (CT = 2).

Net AUC denotes the difference between the AUC of the sample and that of the blank. Said net AUC (Y) is expressed, with respect to the Trolox concentration, as the standard (X), thus obtaining an equation with linear behavior. This equation is used to calculate the mole Trolox equivalents per liter of sample, and the result is expressed as μmol Trolox/g sample, according to Eq. (3).

(3) $$ORAC=\frac{{AUC}_{AH}}{{AUC}_{Trolox}}*\frac{\left[Trolox\right]}{\left[AH\right]}$$

AUC AH: area under the curve in presence of antioxidant, AUC Trolox: area under the curve of Trolox, [Trolox]: Trolox concentration, and [AH]: antioxidant concentration.

##### 2.1.6.3 Monomeric anthocyanin content

To find the monomeric anthocyanin content (MAC), the method described by Lee (2006) was followed by means of a pH differential spectrophotometric analysis [34]. The sample was diluted ten times taking 1 mL of the dilution with 2 mL of 1.0 potassium chloride buffer and 4.5 sodium acetate. This mixture was left to stand for 15 min and then taken to the spectrophotometer at two wavelengths (520 nm and 700 nm). The MAC was expressed in terms of malvidin 3-*O*-glucoside, the most abundant anthocyanin in Isabella grapes [5]. The analysis used a molecular weight of 493 g x mol^−1^ and a molar absorptivity (Ɛ) of 29 500 L x mol^−1^ x cm^−1^, as reported in the literature [35]. The results are expressed in mg of malvidin 3-*O*-glucoside/100 g of sample and are calculated using Eq. (4).

(4) $$MAC\left(\frac{mg\;mal\;3-O\;glucoside}{L}\right)=\frac{\Delta A*MW*DF*1000}{\varepsilon *l}$$

MW: molecular weight of malvidin 3-*O*-glucoside, DF: dilution factor, Ɛ: molar absorptivity of malvidin 3-*O*-glucoside, l: cell optical path (1 cm), and ΔA: change in absorbance (A), determined by Eq. (5).

(5) $$\Delta A={\left(A_{520}\;–\;A_{700}\right)}_{pH\;1.0}\;–\;{\left(A_{520}\;–\;A_{700}\right)}_{pH\;4.5}$$

##### 2.1.6.4 Total flavonoid content

To calculate this content, this study adopted the procedure proposed by Dalli et al. (2021) [36]. First, 200 μl of the previously diluted extract were added to a mixture containing 50 μl of 5% sodium nitrite (w/v) and 1 mL of distilled water. After 6 min of incubation at room temperature, 120 μl of 10% aluminum chloride (w/v) were added to the reaction mixture. The mixture was then subjected to another incubation period of approximately 5 min at room temperature in the dark. Finally, 400 μl of 1 M sodium hydroxide were added. The absorbance of the tubes was measured at 510 nm against the blank. Quercetin was used as the standard to obtain the calibration curve, which was between 10 μg/mL and 100 μg/mL. The flavonoid content was determined and expressed as mg quercetin equivalents/100 g of dried sample. All determinations were conducted in triplicate (6 treatment x 3 replicates, 18 experiments) [36].

#### 2.1.7 Experimental design

##### 2.1.7.1 Two-way factor experimental design

The extraction of the content of the total polyphenol compounds, which are responsible for the antioxidant capacity of Isabella grapes, by USAE technique is affected by various operating parameters. In this parametric study based on a two-way analysis of variance (ANOVA), the independent variables such as solvent and acoustic frequency was considered, in order to analyze their influence on the USAE of the total polyphenol content (TPC) as dependent variable, and a p-value <0.05 was considered as statistically significant criteria. Table 2 describes the factors and levels of the independent variables and the response variable for this phase. Statistical differences between each pair of treatments were evaluated using Tukey's test with a 95% confidence level.

**Table 2:** Factors and levels of the independent variables and the dependent variable for the optimization of the extraction of TPC from Isabella grape by ANOVA.

Statistical Analysis ANOVA	Independent variables		Dependent variable
	Factor	Levels	
First step (Solvent x Frequency)	Solvent	Water: Ethanol (0:1)	Total Polyphenols Content, TPC (mg GAE/g_sample_)
		Water: Ethanol (1:0)	
		Water: Ethanol (0.5:0.5)	
	Frequency	33 kHz	
		40 kHz	

*Each treatment was carried out in triplicate. (6 treatment x 3 replicates, 18 experiments).

##### 2.1.7.2 Polynomial regression model, PRM

According to the results of the two-way factor experiments, the extraction solvent was fixed, and the PRM was used to optimize the USAE conditions to achieve a maximum TPC in the extract. The independent variables were time of the extraction (9 levels), with each extraction time evaluated at two acoustic frequencies in the ultrasonic bath. Each extraction time level is presented in Table 3. The total polyphenols content (TPC, mg GAE/g of Isabella grape) was taken as response for this optimization study. A total of 18 treatments, generated by combinations of the levels of the factors of interest, were replicated four times for a total of 72 experiments. A regression analysis was performed to fit the experimental data to the n-order empirical polynomial model and establish the relationship between the independent variables and the responses. The statistical model was used to determine the optimal conditions for the maximum extraction of TPC, which was then experimentally validated by analysis of variance (ANOVA) (with a 95% confidence interval), in order to evaluate the effect of the following independent variables.

**Table 3:** PRM of USAE of total polyphenol content from Isabella grape.

Statistical Analysis ANOVA	Independent variables		Dependent variable
	Factor	Levels	
Second step (Time x Frequency)	Time (minutes)	15	Total Polyphenols Content, TPC
		30	
		45	
		60	
		75	
		90	
		105	
		120	
		135	
	Frequency	33 kHz	
		40 kHz	

*Each treatment was carried out in quadruplicate (18 treatment x 4 replicates, 72 experiments).

The information was analyzed statistically for both stages using R software (GNU Affero General Public License, version 0.98.1103, available at https://cran.r-project.org).

### 2.2 In vitro evaluation of the antiproliferative effect of isabella grape extract

#### 2.2.1 Cell culture

MCF-7 (ATCC, HTB-22) breast cancer cells were cultured in Dulbecco's Modified Eagle's Medium (DMEM) supplemented with 5% fetal bovine serum (FBS), 100 μg/mL penicillin, and 100 μg/mL streptomycin. The cell cultures were kept under standard conditions in a humidified environment at 37 °C with 5% CO_2_ and 95% air.

#### 2.2.2 Treatment conditions

The cells were seeded at a density of 2.5 × 10^5^ cells/mL under standard culture conditions. After adhesion and exponential growth had been allowed, cells were treated for 24 h with different concentrations of Isabella grape crude extract and processed for biological tests. The stock concentration of the Isabella grape extract employed was 200 mg/mL; for treatments, the preparation of the extract consisted only of a subsequent dilution of optimized extract, but it did not require additional preparation. For all experiments, the final ethanol concentration was 1% or minor.

#### 2.2.3 Cytotoxicity measured by MTT

Cell viability was evaluated using the MTT colorimetric assay on MCF-7 (ATCC, HTB-22™) and MDA-MB-231 (ATCC, HTB-26™) tumor cells and non-tumor HaCaT and L-929 (ATCC, HTB-26™) cells. CCL-1™). Briefly, cells were seeded in 96-well dishes and treated under the culture conditions described above. After 24 h of treatment, 50 μL of MTT (0.5 mg/mL) were added and the mixture was left for 2 h at 37 °C. Finally, 100 μL of isopropanol acid was added to solubilize the formazan crystals. The absorbance at 570 nm was determined in a multimode Varioskan lux multi-plate reader. Data are presented as percentage viability relative to untreated cells, which are 100% viable.

#### 2.2.4 Morphological analysis

The MCF-7 cells were cultured and treated under the culture conditions described above. Afterwards, for the morphological evaluation, these cell cultures were observed and photographed by optical microscopy with a Nikon Eclipse Ti Series inverted microscope.

#### 2.2.5 Cell viability

As a measure of cell viability, cytoplasmic membrane integrity and changes in mitochondrial membrane permeability were evaluated using propidium iodide (PI, Sigma P4170) and 3,3′-dihexyloxacarbocyanine iodide (DiOC_6_, molecular probes D273), respectively. To assess fluorophore incorporation after treatments with Isabella grape extract, the cells were washed twice with phosphate-buffered saline (PBS), trypsinized, sedimented, and stained with 1.5 μg/mL PI and 50 nM DiOC_6_. They were then incubated for 30 min at room temperature. A total of 10 000 events were analyzed by flow cytometry using a BD LSRFortessa cell analyzer, while mean fluorescence intensity (MFI) was calculated using FlowJo software.

#### 2.2.6 Mitochondrial ROS quantification

Relative levels of mitochondrial reactive oxygen species (ROS) were measured using MitoTracker Red (Invitrogen, M7512). After the treatments, the MCF-7 cells were exposed to 3 μM of the dye for 20 min at room temperature. They were then washed twice with PBS and 10 000 events were analyzed by flow cytometry (BD LSRFortessa). The MFI of the MitoTracker™ was calculated using FlowJo.

#### 2.2.7 Hoechst 33 342 staining as an indicator of apoptosis

Apoptosis of MCF-7 cells was quantified by staining with Hoechst 33 342. Breast adenocarcinoma cells were seeded in a 24-well plate at a density of 2.5 × 10^5^ cells/mL and cultured under standard culture conditions for 24 h. After the treatments with Isabella grape extracts, the culture medium was removed and washed with PBS, then the cells were incubated with 50 μl of Hoechst 33 342 at room temperature for 5 min. Apoptotic cells were observed by fluorescence microscopy using the Nikon Eclipse Ti Series inverted microscope. The apoptotic index was calculated as $\frac{number\;of\;apoptotic\;cells}{total\;number\;of\;cells}$ × 100.

#### 2.2.8 Statistical analysis

To compare the treatments, statistical and graphical tests were conducted using R software (GNU Affero General Public License, version 0.98.1103, available at https://cran.r-project.org). First, an exploratory analysis was performed using box plots to observe the trend and dispersion of the data under each of the experimental conditions. Afterwards, one-way ANOVA was carried out to explore the variability between treatments. Values of p ≤ 0.05 were considered statistically significant. All the data represent results obtained from three independent experiments per treatment group.

### 2.3 Molecular docking

Molecular docking simulation studies were conducted using Chimera software (version 1.15). For this purpose, seven phenolic compounds reported in the literature as the most abundant in Isabella grape were used as ligand molecules: *trans*-resveratrol, gallic acid, ferulic acid, caffeic acid, quercetin, catechin, and malvidin [18,37,38]. The chemical structures of these compounds were obtained from the PubChem database in 2D structure and PDB format. Subsequently, an automatic optimization of these ligands was performed with Avogadro software (version 1.2.0).

Similarly, the following proapoptotic and antiapoptotic proteins were selected as receptor molecules: HSP27, Bcl-xL, catalase, TRAIL R1/DR4, Smac/DIABLO, and survivin [28,39]. Their 3D chemical structures were downloaded from the Protein Data Bank and the Protein Data Bank in Europe.

The protein–ligand complex was loaded into Chimera software and prepared for docking using the AM1-BCC algorithm as follows [1]: solvents were removed [2]; hydrogens and charges required to stabilize the molecule were added [3]; protonation states were considered for histidine [4]; ligands and non-protein molecules were removed; and [5] molecular docking was performed using the AutoDock Vina engine. Finally, the results with the highest protein–ligand affinity were evaluated using the Discovery program to analyze the bonds formed in the specific binding site and determine which amino acids formed them.

## 3 Results and discussion

### 3.1 Extraction and extract optimization

#### 3.1.1 Bromatological analysis

Table 4 compares the results of the bromatological analysis of the fresh and dried grapes used in this study after particle-size reduction prior to extraction.

**Table 4:** Bromatological profile^a^ of fresh and dried Isabella grapes.

	Fresh grape (Wet Weight, WW)	Dried grape (Dry weight, DW)
Moisture (%)	84.8 ± 0.041	6.57 ± 0.009
Ash (%)	2.37 ± 0.09	14.6 ± 0.006
Fiber (%)	1.29 ± 0.33	7.94 ± 0.001
Protein (%)	1.09 ± 0.08	6.69 ± 0.007
Fat (%)	0.25 ± 0.01	1.57 ± 0.0003

a The nitrogen-free extract (NFE), consisting mainly of digestible carbohydrates, vitamins, and soluble non-nitrogenous organic compounds was calculated using the equation: NFE (%) = 100-(A + B + C + D + E) where: A = Moisture content (%), B = crude protein content (%),C = crude lipid content (%), D = Crude Fiber Content (%), E = Ash content (%). The result of % NFE for Isabella grapes was 10.20 ± 0.87. [Food and Agriculture Organization of the United Nations. (1996). Rome Declaration on World Food Security. Retrieved from: https://www.fao.org/3/ab489s/AB489S03.htm#ch3].

A literature review that examined publications in this field between 2013 and 2022 [40] did not find any previous studies reporting the bromatological analysis of the *V. labrusca* grape variety. Therefore, Thus, in this study, it was determined that the fiber and ash content of Isabella grapes grown under organic agronomic practices 51.1% and 35.2%, respectively) was higher than the values reported by Bender et al. (2020) [41]*,* who found a fiber percentage of 4.06% ± 1.31 and an ash percentage of 5.14% ± 0.00 in *Vitis vinifera* L dry weight. Fiber content is important due to its capacity to capture water and reduce blood glucose levels [6]. Ash content is related to agricultural practices and depends on the type of manures, fertilizers, and pesticides used in the crop [42]. Llobera and Cañellas (2007) [43] reported an ash percentage of 13.53% ± 0.23 in a grape variety of the *V. vinifera* species called Mango Negro, which is similar to the value reported in Table 4. In the same species, the protein content reported by Balli et al. (2021) [44] was 17%, which is above the values obtained in dry and fresh fruit in other studies [7,44]. In this regard, low protein content is advantageous for the matrix under study because anthocyanins form complexes with proteins, thus reducing the antioxidant capacity [45]. In general terms, the variations in the percentages of nutrients are related to the agroclimatic conditions of the crop [46,47].

#### 3.1.2 Optimization of extraction parameters

The optimized operating parameters were selected considering their impact on the concentration of total polyphenols, according to previous studies on extracts in similar plant matrices [48,49]. The controlled independent variables were type of solvent, acoustic frequency, and extraction time. In addition, the extraction temperature was set to ambient conditions, to protect the thermolabile polyphenols. A plant material: solvent ratio of 1:5 w/v was employed.

##### 3.1.2.1 Solvent selection

Firstly, this study evaluated the effects of the three solvent options (water, water:ethanol, and ethanol) and the simultaneous effects of two frequencies used in the ultrasound bath on the total polyphenol content. The results are summarized in Table 5.

**Table 5:** TPC obtained with different solvents (water and ethanol) and frequencies (33 and 40 kHz).

Solvent	Frequency kHz	TPC mg GAE/g _sample_
Water: Ethanol (1:0)	33^a^	6.82 ± 0.65^a^
Water:Ethanol (0:1)	33^a^	6.71 ± 0.39^a^
Water:Ethanol (0.5:0.5)	33^a^	15.42 ± 0.53^b^
Water: Ethanol (1:0)	40^a^	7.21 ± 1.22^a^
Water:Ethanol (0:1)	40^a^	6.13 ± 0.08^a^
Water:Ethanol (0.5:0.5)	40^a^	14.63 ± 1.78^b^

TPC values are expressed as gallic acid equivalent (GAE), on a dry basis and reported as mean value and standard deviation (SD), n = 3 (6 treatments x 3 replicates = 18 experiments). Different superscript letters in the same column indicate significant differences (p < 0.05).

ANOVA shows that there are significant differences between each of the solvent types (*P*-value: 2.97e-10), with the water:ethanol mixture (0.5:0.5 v/v), being the treatment that differed from the extractions performed with only water or ethanol according to Tukey's test (See *P*-values in Table 1S). The binary mixture was more efficient in terms of the amount of TPC extracted, presenting extracted polyphenol contents above 50%, in the experiments performed at both 33 kHz and at 40 kHz (Table 5). For this reason, the water:ethanol mixture (0.5:0.5 v/v) was selected as the operating parameter for the optimization of the TPC extraction method from Isabella grapes.

The superior performance of the water:ethanol (0.5:0.5 v/v) solvent system could be due to the water:ethanol mixture improving the relationship between the surface tension of both liquids, specifically by decreasing the tension provided by water (g_20°C_: 72.9 mN/m) with the ethanol (g_20°C_ = 22.1 mN/m), as well as the balance of the density provided by each solvent (d_water_ = 1.000 g/m and d_ethanol_ = 0.879 g/mL). These final properties of the mixture facilitate its penetration into the matrix. Similarly, its high dielectric constants (k_ethanol at 25°C_ = 24.6, k_water_ _at 25°C_ = 77.46) favor the solvation of polar compounds, such as the polyphenols present in Isabella grapes, due to their ability to decrease the intermolecular energy at low temperatures (24 ± 1 °C in this study), thus protecting thermolabile compounds [50]. Due to its properties, the protic solvent mixture mentioned above can achieve a better extraction rate of phenolic compounds than pure solvents [9,[51], [52], [53]]. Furthermore, this hydroethanolic mixture is environmentally friendly and safe for food or pharmaceutical applications because it poses a low risk of toxicity [54].

**Fig. 2:**
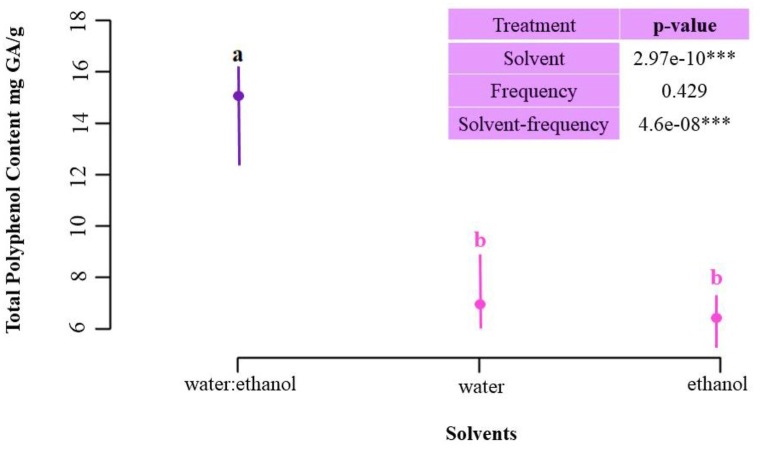
ANOVA of the extraction solvent with three levels (water, ethanol, and water:ethanol) and the TPC as the response variable. Different superscript letters (a,b) indicate significant differences (p < 0.05 by Tukey's test).

With regard to the evaluation of the effect of acoustic frequencies on the TPC extraction capacity of Isabella grapes using USAE, there were no significant differences (*P*-value: 0.429) in the TPC results obtained at the levels studied (33 and 40 kHz) Fig. 2. However, the interaction between the type of solvent and extraction frequency did show significant differences (*P*-value: 4.6e-08). For this reason, the most optimal condition could not be selected at this statistical analysis and the effect of the frequency over the course of the PRM was re-evaluated, together with the optimization of the extraction time, as shown in section 2.4.

##### 3.1.2.2 PRM optimization of extraction conditions (time and acoustic frequency)

The evaluation of the effect of the simultaneous variation of the independent variables of acoustic frequency (33 and 40 kHz); and extraction time, from 15 min to 135 min with intervals of 15 min, on the content of total polyphenols in the Isabella grape, presented a non-significant linear component. For this, a polynomial model was needed to establish the regression equations that allowed optimization of the operational parameters studied, so that they would lead to the identification of the maximum TPC in the Isabella grape extract obtained by ultrasonic bath. In addition, the predicted values from the measured values were defined for each treatment of the experimental design, and are shown in Table 6.

**Table 6:** Measured and predicted TPC values, determined for the simultaneous variation of the independent variables of acoustic frequency (33 and 40 kHz) and extraction time from 15 min to 135 min with intervals of 15 min. Extractions were carried out under sonication of 100 W, at 24 °C ± 1, with water:ethanol (0.5:0.5 v/v) and L/S (5:1).

Treatment	Independent variables		Response Variables		Treatment	Independent variables		Response Variables	
			(TPC mg GAE/g db)					(TPC mg GAE/g db)	
	*X*1: Time	*X*2: Frequency	Measured	Predicted		*X*1: Time	*X*2: Frequency	Measured	Predicted
1	15	33	9.0^e^	9.3	10	15	40	5.9^c^	6.5
2	30	33	11.6^d^	11,.1	11	30	40	8.7^a^	7.3
3	45	33	13.3^c^	13.2	12	45	40	7.3^b^	8
4	60	33	14.6^b,c^	15.2	13	60	40	8.4^a^	8.7
5	75	33	17.1^a^	16.5	14	75	40	8.8^a^	8.9
6	90	33	15.6^a,b^	16.7	15	90	40	8.9^a^	8.8
7	105	33	16.3^a^	15.3	16	105	40	8.3^a^	7.9
8	120	33	11.8^d^	11.9	17	120	40	6.2^c^	6.3
9	135	33	5.8^f^	5.9	18	135	40	3.6^d^	3.7

TPC values are expressed as gallic acid equivalent (GAE), on a dry basis (18 treatments x 4 replicates = 72 experiments). Different superscript letters in the same column indicate significant differences (p < 0.05 by Tukey's test).

The effects of the main factors of frequency and time were significant (*P*-value_33kHz_: 5.79e-05 and *P*-value_40kHz_: 0.0033) and there was evidence of a variation of the TPC depending on both factors. To explain the effects of the factors, a polynomial model of order 2 was necessary for the 33 kHz frequency, defined by Eq. (6), and of order 3 for the frequency of 40 kHz framed in Eq. (7).

(6) $$\overset{\wedge }{\overset{︷}{TPC}}=12.7808-0.6931\times Time-9.7933\times {Time}^{2}$$

(7) $$\overset{\wedge }{\overset{︷}{TPC}}=7.3334-1.8132\times Time-4.2393\times Time2-0.9884\times {Time}^{3}$$

**Fig. 3:**
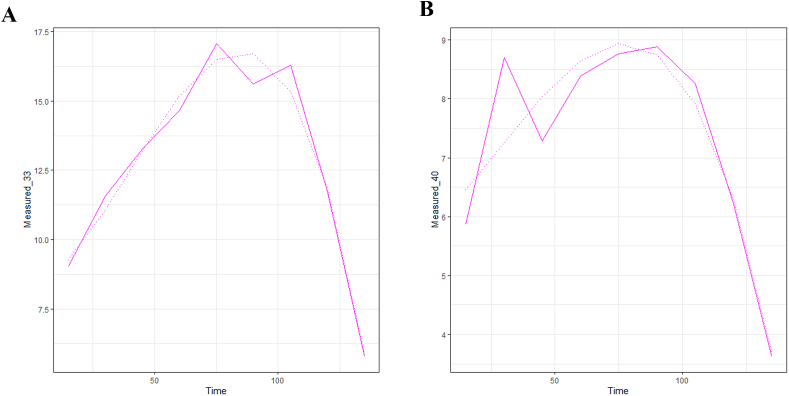
Non-linear regression between TPC and t values during extraction at 33 kHz (3 A plot) and 40 kHz (3 B plot) from Isabella grape; L/S = 5 mL/g and water:ethanol (0.5:0.5 v/v). Extractions were carried out under sonication (100 W) and at 24 °C ± 1.

Fig. 3 shows the nonlinear behavior corresponding to the polynomial regression models explaining the trend of total polyphenol content extracted from Isabella grapes under the optimized conditions of the acoustic frequencies evaluated according to the method described in section 2.1*.* Fig. 3A presents the extraction behavior at 33 kHz and Fig. 3B at 40 kHz.

The assumptions were validated, fulfilling in both models the normality for residuals, equal variances, and random behavior of the residuals. In terms of the measure of the proportion of the variance of the two models obtained, in the analysis it is possible to obtain an adjustment evaluated by means of the squared coefficients of connections (R2). This, for the extractions of TPC at 33 kHz and for those of TPC at 40 kHz, were 97.18% and 87.25%, respectively, with a p value < 0.05, indicating statistically significant agreement between observed and predicted responses. In addition, it can be inferred that, with the frequency of 33 kHz, there is a greater range to identify better behavior of the TPCs.

**Fig. 4:**
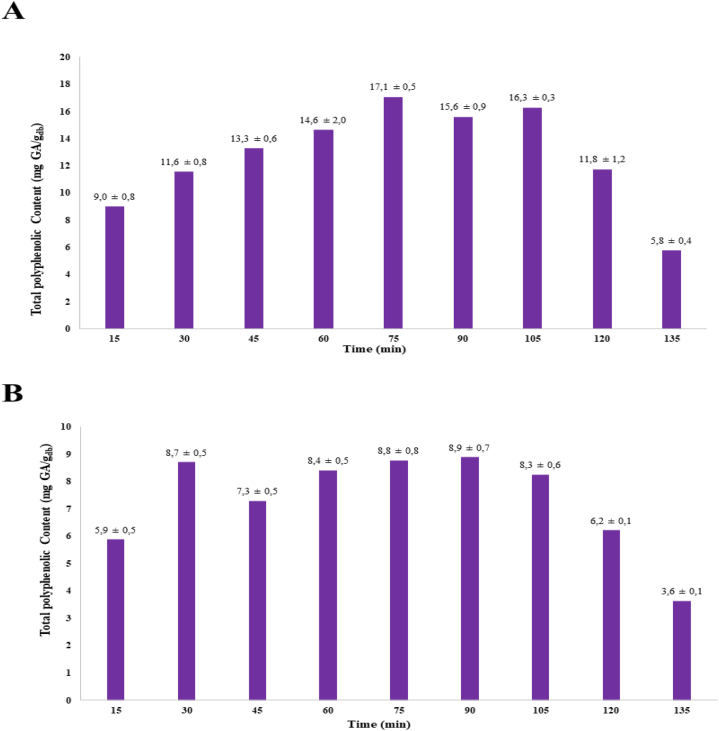
Extraction time variation every 15 min until reaching 135 min. (A) and (B) show the TPC values, which are expressed as gallic acid equivalent (GAE), on a dry basis and reported as mean value and standard deviation (SD) obtained from Isabella grape at frequencies of 33 and 40 kHz, respectively.

The optimal extraction time was selected by following the procedure described in the *Materials and methods* section above. Fig. 4A presents the extraction time variation at a frequency of 33 kHz. The two highest TPC values are observed at 75 and 105 min. Likewise, Fig. 4B shows said variation at a frequency of 40 kHz, with a maximum TPC value at 30 min.

**Fig. 5:**
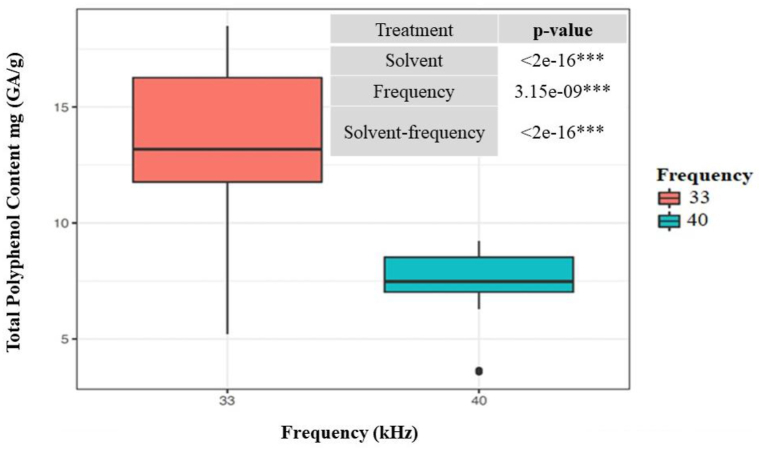
Box plot of the ANOVA test that compares the effects of the 33-kHz and 40-kHz frequencies on the TPC from Isabella grape on a dry basis as the response variable. Inset: p-values of the time and frequency parameters and the time–frequency interaction.

The results of the ANOVA test revealed significant differences between the TPC averages evaluated at the two frequencies, with a 95% confidence level (See *P*-values in Fig. 5). The 33-kHz frequency displayed higher efficiency in the extraction yield than its 40-kHz counterpart, as shown in Fig. 5. As a result, the ultrasound operation at 33 kHz is the optimal frequency for the extraction of polyphenols present in the sample under analysis.

The acoustic frequency at which it was possible to determine the maximum value of the total polyphenol content (33 kHz), led to an average TPC value that was 37.12% higher than previous studies on *V. labrusca*, which obtained 11.01 mg GAE/g by percolation [55]. Other results published for *V. vinifera* pomace extracted by ultrasound without extenuation (16.1 mg GAE/g) [29] are 8.1% lower than those found in this study without extenuation. These differences can be attributed not only to the genetic information of the material that was evaluated, but also to the operating conditions. Low frequencies and powers have been proven to have a positive effect on extraction yield. Conversely, high powers and frequencies can promote the generation of free radicals in the medium, inducing the degradation of aromatic or unsaturated compounds present in the matrix [56]. The power setting employed in this study was 100 W, which is low considering that polyphenol extractions have been reported in a range between 100 W and 450 W [9,57]. In view of the favorable results obtained at low powers, this parameter was kept fixed.

Additionally, statistical analysis confirmed that the extraction was most efficient at 33 kHz, which was selected as the optimal frequency. The TPC values obtained at 40 kHz could be affected by the formation of microbubbles during the extraction, which collapse and generate hydrogen and hydroxyl atoms in the aqueous solution, causing the decomposition of solvents and solutes and the polymerization and degradation of phenolic compounds [58]. Another study reported a higher TPC using extraction parameters of 20 kHz and 150 Watt for 30 min, obtaining up to 105.81 mg GAE/g of sample from *Vitis vinifera* seeds [9]. This result, superior to those of the present study, may be due to the use of methanol as solvent, and to the fact that the extract was concentrated by rotaevaporation, which promotes a higher TPC yield. In addition, there may be an influence of the solvation capacity of methanol, especially on anthocyanins [30]. However, methanol was not considered as a solvent in this study since this is a result for possible applications in food, cosmetics, or pharmaceuticals [11].

In other studies, grape seeds have been shown to contain larger amounts of polyphenols than other fruit matrices (skin, pulp, and stem) [43,59,60] This is because sugars, organic acids, salts, and proteins found in the matrices react with the phenolic compounds and, therefore, affect their antioxidant capacity [17]. However, Gomes et al. (2019) demonstrated that quercetin and diglucoside anthocyanins (the latter mostly found in the skin) have the better bioaccessibility than other phenolic compounds found in grapes, which led them to conclude that Isabella grape has potential as a chemopreventive food [61]. Hence, it is important to study an extract obtained from the whole fruit.

Table 6 shows the TPC values determined during the extraction every 15 min until reaching 135 min. Four groups can be identified (marked using the superscripts ^a^, ^b^, ^c^, and ^d^), which comprise the times that did not differ significantly from each other with respect to the response variable (i.e., TPC) (See *P*-values in T2S). The highest TPC values ranged between 15.6 and 17.1 mg GAE/g and were achieved at 75, 90 and 105 min. These times were analyzed to select the optimum, which corresponds to 75 min. In terms of efficiency, evaluating the processing time is a relevant step in the implementation of the extraction method because it determines the period needed to extract the highest TPC. Additionally, the compound of interest could be degraded by prolonged exposure to solvents. In this study, the optimal extraction time was 75 min. However, Moldovan et al. (2020) analyzed two shorter times (15 and 30 min, at 40 °C with a 70% ethanolic solution) to extract phenolic compounds from grape juice and obtained TPC results 12% below the present study [62].

Although similar yields in shorter times have been reported for *V. vinifera*, this species has a better concentration of these compounds and, consequently, a higher yield [29]. This behavior was also evidenced in the work reported by Ref. [63] who carried out the regression study for the optimization of the extraction time of polyphenols and pigments from solid onion residues, finding a polynomial regression model similar to that of the present study. However, in that study it was not possible to demonstrate the fall of the TPC because the extraction time only reached 60 min, less than half the time evaluated in this investigation. It should be noted that the diffusion of the solute in the solvent in plant matrices does not occur in a homogeneous or specific way for polyphenols, because, as is known, plant cells have a very diverse set of molecules in addition to polyphenols, which can interact with each other, leading to possible degradation of the TPC. In this sense, the behavior can be explained by the selectivity of the solvent, since during the extraction time molecules related to the polarity of the solvent may have been mixed with the polyphenol-rich ethanolic extract, generating a reaction that would lead to the degradation of the solvent. Thus, by continuing with this extraction, the TPC may have increased again because there were still polyphenols inside the plant material and saturation of the solvent had not yet occurred. On the other hand, regarding the TPC drop, in another study it was found that throughout the extraction time, the sonication energy decreases, generating loss of energy and thus decreasing the extraction yield, behavior that could explain why the concentration of polyphenols decreased in the Isabella grape extract after 75 min, with the drop becoming sharper after 105 min [64].

In terms of extraction time efficiency, it is essential to understand the type of phenolic compounds and the matrix under study. For example, Tao et al. (2014) found a maximum TPC value of 24.42 mg/g after submitting a commercial grape sample to ultrasound for 80 min without extenuation, but using 40 °C. Although this temperature decreases the surface tension of the solvent, it may induce the degradation of anthocyanins, which are important due to their antioxidant capacity [65]. Therefore, sample extenuation has been used to improve the resulting TPC yield. Hence, this study obtained a total of 43.14 ± 5.00 mg GAE/g of sample, which is similar to the value reported for *V. vinifera.* Regarding *V. labrusca*, Prieto et al. (2011) and Burin et al. (2014) found a maximum TPC yield of 11.01 mg GAE/g and 0.56 mg GAE/g of sample, respectively, using ultrasound-assisted extraction, but without extenuation [55,66]. This last result is below that achieved in this study without extenuation, which was 17.08 ± 0.5 mg AG/g of sample, but it was not conclusive evidence in favor of continuing to use the proposed method of extraction without taking advantage of extenuation. The ability to considerably increase the content of total polyphenols extracted from the *Vitis labrusca* variety by up to approximately 64%.

#### 3.1.3 Antioxidant capacity and total anthocyanin content of the optimized extract

Under the optimized operating parameters, the sample was extenuated, avoiding solvent saturation, and the yield was improved, with TPC extraction increasing by 39.7%, for a total of 43.14 ± 5.00 mg GAE/g of sample. Table 7 shows the characterization of the extract, i.e., its profile in terms of functional properties associated with antioxidant capacity and anthocyanin content.

**Table 7:** Optimal extraction parameters (frequency, time, and solvent) and resulting TPC (mg GAE/g), antioxidant capacity (ORAC), and MAC of the extenuated sample.

Extraction parameter	Optimal value	TPC mg GAE/g of sample_dw_	Antioxidant capacity (ORAC) μmol of Trolox/g of sample_dw_	MAC mg of malvidin-3-glucoside/100 g of sample
Frequency (kHz)	33	43.14 ± 5.00	293.22.1 ± 34.73	17.69 ± 2.59
Time (min)	75			
Solvent	Water:ethanol (0.5:0.5)			

The antioxidant capacity of *V. labrusca* achieved here using the optimized extraction method (i.e., 293.23 ± 34.73 μmol of Trolox/g of sample) is higher than those reported in other studies on grapes [67,68]. For instance, Bender et al. [69] reported values of 164.0 ± 1.4 μmol of Trolox/g of sample in *V. labrusca* grape pomace after an extraction time of 15 min in an ultrasonic bath. Another study on a commercial variety of *V. vinifera* [70] obtained an antioxidant capacity 81% lower than that found here. This difference may be explained by the traditional extraction method used in said study because it is known that yields are higher when ultrasound-assisted extraction is implemented [71]. Ramón and Gil-Garzón reported a TCP 93% lower than that obtained here, which explains the lower antioxidant capacity they found [40]. These results prove the importance of time optimization, sample extenuation, and entire matrix evaluation.

Li et al. (2019) found an antioxidant capacity of 190.57 μmol Trolox/g in the skin of the Muscat Kyoho grape variety and only 16.24 μmol Trolox/g in its pulp [72]. Therefore, it is important to evaluate the content of monomeric anthocyanins because this group of polyphenols is abundant in the skin of Isabella grapes [17]. In this regard, Decendit et al. (2013) found that the most common anthocyanin in grapes is malvidin, which is described as an anti-inflammatory agent with no detectable toxic effects [73]. Bender et al. (2020) managed to extract up to 13.1 mg malvidin-3-glucoside/100 g from Cabernet Sauvignon grapes using an extraction technology called microwave hydrodiffusion and gravity. Although they applied high powers and frequencies that produced a higher energy expenditure, they obtained an amount of malvidin lower than that achieved in this study (i.e., 17.69 ± 2.59 mg malvidin-3-glucoside/100 g). Meanwhile, Porto et al. (2013) obtained 2.2 mg malvidin-3-glucoside/g from the seeds of the *V. vinifera* variety; however, they used petroleum-derived organic solvents, such as hexane, which can improve extraction yields but are toxic to human health. In addition, prior to the ultrasound-assisted extraction, they performed a Soxhlet extraction, which can be inconvenient for industrial scale-up [9,41].

In this study, a total flavonoid content of 18.90 ± 0.50 mg quercetin/g dry weight was found in the optimized extract. This result is similar to that reported by Souza et al. (2014), who obtained 18.90 ± 0.60 mg quercetin/g dry weight mixed with 20% maltodextrin in a microencapsulation of anthocyanins from *V. labrusca* [74]. Nevertheless, said content is lower than that reported by Ribeiro et al. (2015), who evaluated different grape species and found that *V. labrusca* had the highest flavonoid content, with a maximum value of 21.57 ± 0.10 mg quercetin/g [5]. However, they used shaking extraction for 24 h, which makes the method difficult to scale up in the future.

### 3.2 In vitro evaluation of the antiproliferative effect of isabella grape extract

Breast cancer has been the most diagnosed type of cancer worldwide in recent years, and the number of new cases is expected to grow in the short term [75]. Although chemotherapy is the most widely used treatment for this disease, it has harmful side effects, which has motivated the exploration of natural compounds as adjuvant agents that can offer health benefits while ensuring low toxicity levels [20]. In this study, *V. labrusca* extract showed a high polyphenols content and antioxidant capacity. Therefore, its cytotoxic activity was evaluated *in vitro* on breast cancer cell lines, and non-tumoral cells.

#### 3.2.1 Cytotoxic studies

**Fig. 6:**
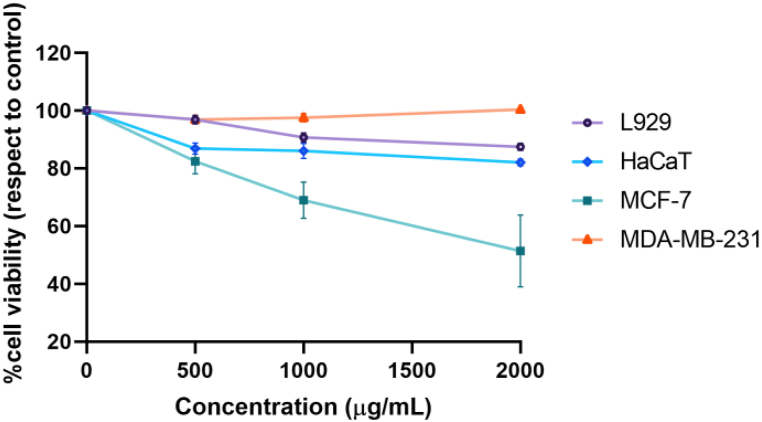
Cytotoxic effect of treatment with Isabella grape extract. Cells were treated for 24 h with different concentration of optimized extract, and cytotoxic effect was determinate by MTT assay. The values are the means of three independent experiments with each treatment concentration.

The cytotoxicity of *V. labrusca* extract was evaluated using MTT assay in the breast cancer cell lines MCF-7 and MDA-MB-231 and in the non-tumoral cell lines HaCaT and L-929 (Fig. 6). Table 8 shows the half-maximal inhibitory concentration (IC_50_) at 24 h of treatment using different concentrations of the grape extract. The results revealed an IC_50_ value of 2189 μg/mL for MCF-7, and 159 256 μg/mL for MDA-MB-231. For non-tumoral cells, the IC_50_ values were 9893 μg/mL and 22 615 μg/mL for HaCaT and L-929, respectively.

**Table 8:** IC_50_ values of Isabella grape extract on tumoral (MCF-7 and MDA-MB-231) and non-tumoral cells (HaCaT and L-929).

	MCF-7	MDA-MB-231	HaCaT	L-929
IC_50_ values (μg/mL)	2189	159 256	9893	22 615

Table 8 shows that the lowest IC_50_ value for evaluated cell lines was obtained for MCF-7 cells. To confirm that the *V. labrusca* extract has an anti-cancer effect instead of a toxic effect, selectivity index (SX) was calculated as $SX=\frac{{IC}_{50}\;HaCaT}{{IC}_{50}\;MCF-7}\;x\;100$. SX value > 100 denotes that the cytotoxic effect is more selective in cancer cells [76]. For our data, the value obtained was 451.9, which is indicative of selective toxicity to breast cancer cells.

To investigate the mode of action of *V. labrusca* extract, we continued our experiments on the MCF-7 cells by evaluation of morphological changes, membrane cell permeability, mitochondrial potential, ROS generation, and apoptosis induction.

#### 3.2.2 Morphological analysis

**Fig. 7:**
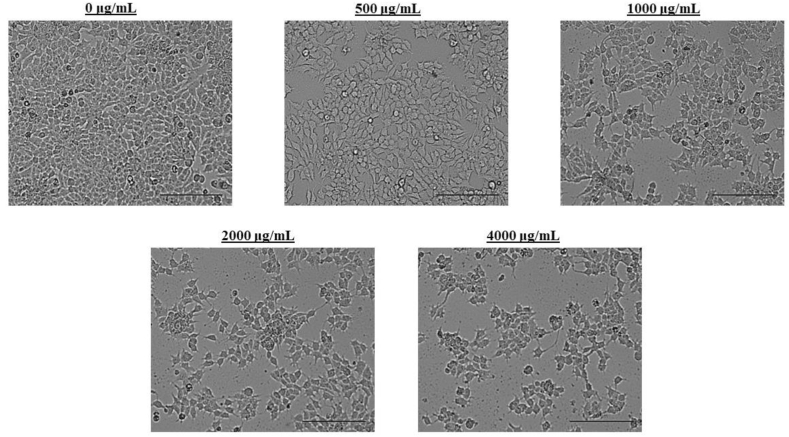
Morphological characterization of MCF-7 cells after treatment with Isabella grape extract at different concentrations for 24 h and observation by optical microscopy for morphological analysis. The scale bar is 50 μm.

The qualitative description of cell morphological changes was made according to International Organization for Standardization 10 993–5 [77]. Fig. 7 is composed of representative photographs of the cell cultures, in which untreated MCF-7 cells show an epithelial morphology (typical of this cell line) and extensive monolayer growth on the surface, in addition to the presence of round and bright cells (mitotic cells) adhered to the substrate. Concentrations of 500 and 1000 μg/mL of grape extract induced changes in cell morphology: cells appear elongated, the number of mitotic cells decreases, and there is cell debris. With the 2000 and 4000 μg/mL treatments, there is destruction of the cell monolayer, cell debris, little presence of mitotic cells, and less than 50% of the initial cell population.

#### 3.2.3 Effect of Isabella grape extract on cell viability

**Fig. 8:**
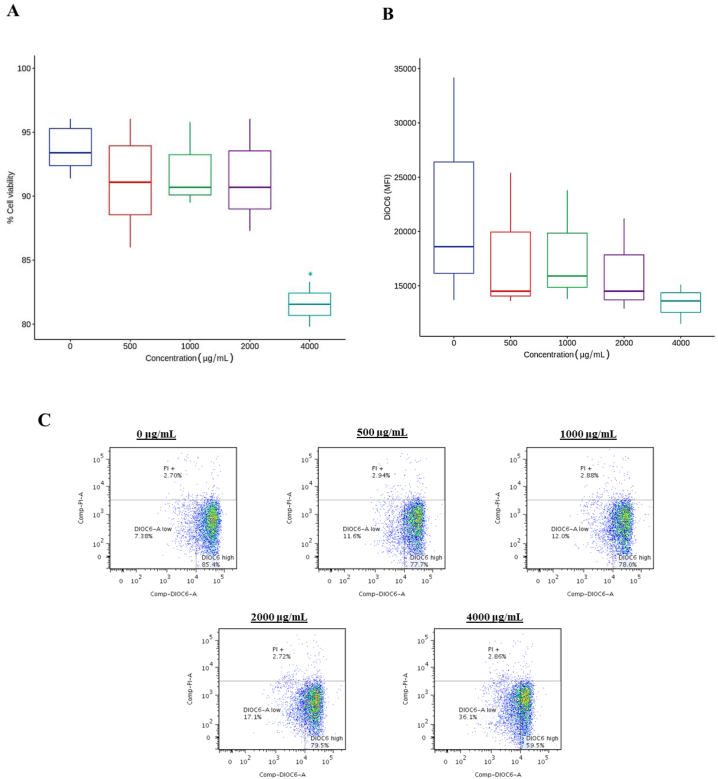
Effect of Isabella grape extract on the viability and mitochondrial integrity of MCF-7 cells. The cells were treated with different concentrations of the extract for 24 h and subsequently analyzed by flow cytometry. (A) Cell viability measured by PI incorporation. (B) DiOC_6_ uptake as a measure of mitochondrial membrane polarization. (C) Scatter plot detailing the behavior of MCF-7 cells as the concentration of Isabella grape extract increased. The values are the means of three independent experiments with each treatment concentration, which were compared using Dunnett's method. *p ≤ 0.05.

Fig. 8A shows the viability of MCF-7 cells after 24 h of treatment with Isabella grape extract. The results evidence a dose-dependent decrease in cell viability, with a significant difference at the 4000 μg/mL concentration. Changes in mitochondrial membrane permeability and potential were evaluated as indicators of cell viability. DiOC_6_ dye accumulates in the mitochondrial matrix and is released into the cytosol when membrane depolarization occurs [78]. Fig. 8B shows a decrease in dye uptake as the extract concentration increases. Finally, Fig. 8C presents a representative dot plot detailing the behavior of MCF-7 cells as the concentration of delivered Isabella grape extract is increased.

#### 3.2.4 Prooxidant effect of isabella grape extract on MCF-7 cells

**Fig. 9:**
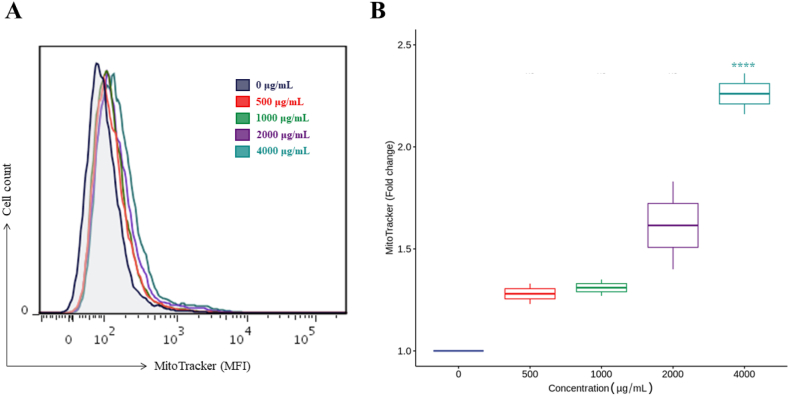
Mitochondrial ROS production quantification. The cells were treated with different concentrations of the extract for 24 h and subsequently analyzed by flow cytometry. (A) Histogram shows higher MitoTracker fluorescence intensity in MCF-7 cells as the extract concentration was increased. (B) Box plot presents the means of three independent experiments with each treatment concentration. The values are the means of three independent experiments with each treatment concentration, which were compared using Dunnett's method. ****p ≤ 0.0001.

Mitochondrial ROS result from the aerobic metabolism of cells. When exposed to oxidative stress, the latter accumulate oxygen radicals in the mitochondria, causing damage at the cellular level [79]. Fig. 9 shows higher concentrations of mitochondrial ROS in MCF-7 cells as the concentration of the extract was increased. Significant differences occurred from 4000 μg/mL. Fig. 9A shows a representative histogram of the increase in MitoTracker fluorescence intensity in MCF-7 cells treated with Isabella grape extract. Likewise, Fig. 9B presents a boxplot with the means of three independent experiments with each treatment concentration.

#### 3.2.5 Apoptotic effect of isabella grape extract on MCF-7 cells

**Fig. 10:**
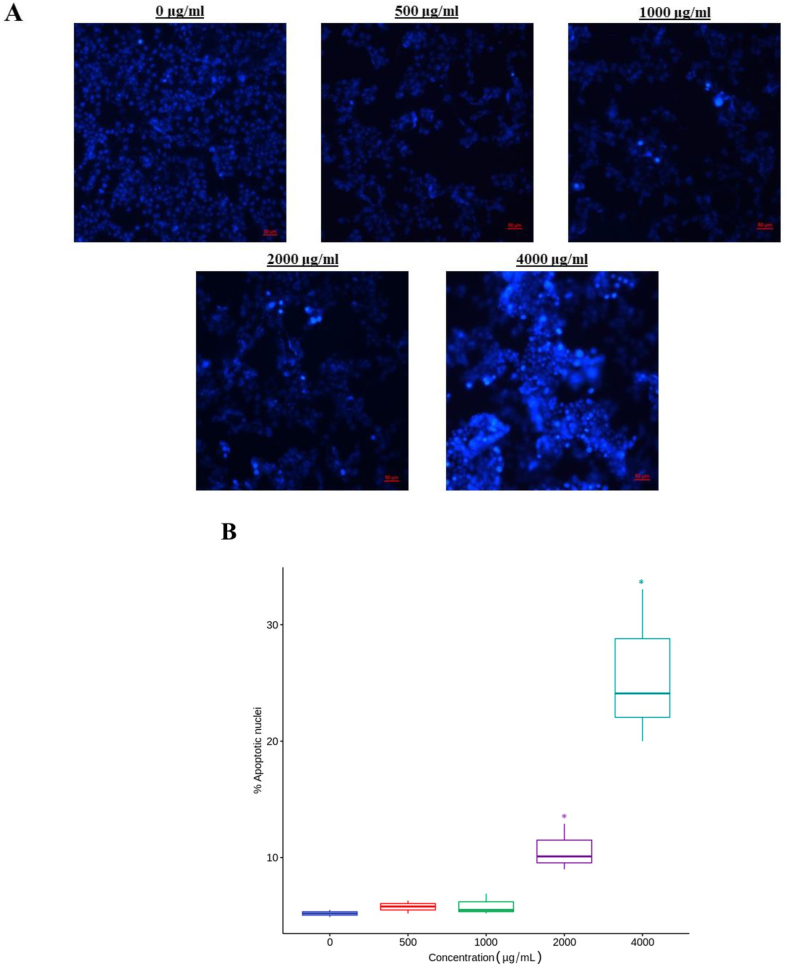
Apoptosis detected by Hoechst 33 342 staining. The cells were treated with different concentrations of grape extract for 24 h and subsequently analyzed using fluorescence microscopy. (A) Representative images of cells treated with grape extracts at different concentrations and stained by Hoechst. The scale bar is 50 μm. (B) Percentage of apoptotic nuclei detected after staining with Hoechst, at least 500 cells were analyzed per assay. The values are the means of three independent experiments with each treatment concentration, which were compared using Dunnett's method. *p ≤ 0.05.

Fig. 10 presents the results obtained after treating MCF-7 cells for 24 h with Isabella grape extracts and staining with Hoechst. This intercalating agent has an affinity for regions of DNA rich in thymine and adenine and shows greater fluorescence intensity when there is chromatin condensation, one of the characteristics of the programmed cell death process [80]. Fig. 10A shows how, as the concentration of the extract increases, the number of cells with chromatin condensation increases proportionally. The apoptosis index was calculated as the number of cells with apoptotic nuclei/total number of cells × 100. The data obtained are presented in Fig. 10B, where the increase in cells with apoptotic nuclei can be quantitatively evidenced as the concentration increases of the treatment with Isabella grape, the results being significant from the dose of 2000 μg/mL.

The most abundant phenolic compound in the extract obtained here was malvidin, which is an anthocyanin. Anthocyanins regulate the gene expression of multiple signaling pathways; inhibit cell proliferation, inflammation, and angiogenesis; and promote apoptosis and cell differentiation [81]. Guo et al. (2020) demonstrated that black raspberry anthocyanins regulate the pathways related to miR-483–3p/DKK3/Wnt/β-catenin and miR-338–5p/SIRT1, thus affecting colorectal cancer progression [82]. In their study, Su et al. (2018) concluded that blackberry anthocyanins induce apoptosis and autophagic cell death in thyroid cancer cells [83]. In addition, two other phenolic compounds (flavonols), i.e., catechin and quercetin, were found in the extract analyzed here. According to the literature, they have a significant presence in Isabella grapes and could be responsible for the cytotoxicity generated in MCF-7 cells [5,38,66,69].

Regarding breast cancer, the anthocyanins of Isabella grape and cherry have been reported to suppress intracellular ROS generation [84,85]. An extract enriched with flavonoids and cherry anthocyanins evaluated by Lage et al. (2020) on MDA-MB-453 cells reduced cell proliferation and downregulated the PI3K/Akt/mTOR and Sirt1/survivin pathways, leading to the induction of apoptosis through PARP cleavage [84]. Flavonols exert a cytotoxic effect on tumor cells by significantly inducing apoptosis markers such as cleaved caspase-3/7/8, PARP, and Bax/Bcl-2 [86]. In skin cancer, it has been demonstrated that fisetin flavonoids inhibit molecules such as CDK2, phosphorylated c-Kit and its downstream effectors including Akt, mTOR, p70S6K, p90RSK, Stat3, and MAPK (ERK1/2) [86]. Although it has been reported that flavonoids decrease the expression and activity of catalase, which is related to a loss of antioxidant capacity [87,88], according to Lin et al. (2016) and Khan et al. (2021), they are potent free-radical scavengers [[88], [89], [90]].

An antioxidant agent is one that prevents the oxidation of molecules within a cell by removing electrons or hydrogen from a substance. Because radicals are reactive, they initiate the chain reaction simultaneously, which can lead to cell damage or death [91]. However, there is scientific evidence that suggests that phytochemicals also elicit a prooxidant behavior related to anticancer effects. Compared to their normal counterparts, cancer cells have elevated levels of ROS and an altered redox state to maintain their malignant phenotypes. For this reason, cancer cells are more vulnerable to increased ROS production induced by prooxidant agents than normal ones [92]. The results reported in this study show that Isabella grape extract induced prooxidant activity in MCF-7 cells by elevating mitochondrial ROS levels, which is possibly associated with the observed loss of cell viability (Fig. 9). In line with our research, Lee et al. (2013) used coumestrol extracted from leaves of *Glycine* max to treat MCF-7 cells. Their results revealed the promotion of cellular senescence through the p53-p21 Cip1/WAF1 pathway, induced by high ROS production [93]. Khan et al. (2020) proposed that natural dietary products (in particular, plant-derived phenolic compounds) can take advantage of elevated copper levels in the tumor microenvironment and promote ROS generation, leading to selective DNA damage of cancer cells [94]. Goleva et al. exposed A549 human lung carcinoma cells (another cell model) to curcumin treatments; as a result, the mitochondrial ROS production grew, which was associated with increased Ca^2+^, loss of membrane potential, and activation of caspase-9 and caspase-3, ultimately leading to cell apoptosis [92]. Heo et al. (2018) treated A375 human melanoma cells with phytochemicals, which resulted in inhibition of cell growth mediated by activation of the apoptotic ROS-p38-p53 pathway by increasing the level of phosphorylated p38, MAPK, and p53 [95].

Mitochondrial membrane potential is an indicator of the energy state of the mitochondria and the cell. It can be used to assess the activity of mitochondrial proton pumps, electrogenic transport systems, and mitochondrial permeability [96]. MDA-MB-231 and T47D breast cancer cells have shown a decrease in mitochondrial membrane potential (in a dose-dependent manner) when treated with *Bulbine frutescens* extracts [23]. The results in Fig. 8B indicate mitochondrial membrane depolarization caused by the Isabella grape extract. Several studies have correlated mitochondrial depolarization with the induction of apoptosis through caspase activation [21,97]. Likewise, excessive ROS production and altered mitochondrial membrane potential are associated with the initiation of apoptosis in breast cancer cells [23,98]. Fig. 10 shows the percentage of apoptotic nuclei observed through Hoechst 33 342 staining. Healthy cells typically have spherical nuclei with uniform DNA distribution, whereas the DNA is condensed during apoptosis [80]. Consequently, nuclear condensation can be observed as more fluorescent nuclei after staining. In our results, increased fluorescence intensity was directly proportional to the increased concentration of the extract, which could suggest cell death by apoptosis. Nevertheless, more specific studies are necessary to confirm this type of cell death. Nevertheless, more specific studies are necessary to confirm this type of cell death.

### 3.3 Molecular docking

The *in vitro* experiments have shown that the grape extract induces cytotoxicity associated with its effects on the mitochondria and nuclear condensation, which could be related to apoptosis. As a result, further computational studies were proposed to explore the potential mechanism of apoptosis-induced death. In this way, *in silico* assays were carried out to investigate the molecular interactions between some of the phenolic compounds reported for Isabella grape and the apoptosis pathway proteins. As ligands were selected seven phenolic compounds reported previously in the literature as the most abundant in Isabella grape and known to induce apoptotic cell death in various cancer models: *trans*-resveratrol, gallic acid, ferulic acid, caffeic acid, quercetin, catechin, and malvidin. The apoptosis proteins selected were Hs27, Bcl-XL, catalase, TRAIL R1/DR4, Smac/DIABLO, and survivin. Heat shock protein (Hsp27), one of the members of the sHsp family, is a cell survival regulatory factor associated with chaperone functions. It is involved in different signaling pathways, including the regulation of apoptosis through the inhibition of caspases and binding to cytochrome *c* [99]. B-cell lymphoma-extra-large (Bcl-xL), a member of the Bcl-2 family of proteins, is a mitochondrial transmembrane protein that plays a key role in the regulation of the intrinsic apoptosis pathway and is overexpressed in different types of cancer [100]. Catalase is a common enzyme found in all living organisms, that breaks down hydrogen peroxide (H_2_O_2_) into water and oxygen, reducing oxidative stress [101]. In most cases, this protein has antiapoptotic activity through scavenging ROS [102]. TRAIL R1/DR4 are cell death receptors that induce apoptosis by both intrinsic and extrinsic pathways [103].

The importance of TRAIL for clinical strategies lies in its capacity to target tumor cells without damaging their non-malignant counterparts [104]. Another protein released by mitochondria and involved in the regulation of apoptosis is the second mitochondria-derived activator of caspase (Smac), also called DIABLO. Smac negatively regulates different proteins belonging to the IAP (inhibitor of apoptosis proteins) family and is, therefore, considered a key regulator of apoptosis in mammals [105]. Survivin/BIRC5, another inhibitor of various IAPs, is absent in differentiated tissues but highly expressed in developing tissues, transformed cells, and most human tumors. Because of its specificity as a tumor antigen, survivin represents a very promising therapeutic target [106].

Table 9 summarizes the results obtained from the protein–ligand docking simulated in AutoDock Vina software. The relationship between the phenolic compounds evaluated here and the apoptosis-related proteins was measured by means of a dimensionless number calculated by the program. This number denotes a dissociation constant in the interactions or bonds formed by the protein–ligand complex that is inversely proportional to the sign of the dimensionless number. Therefore, the more negative the number of the dissociation constant, the more stable the interactions. In other words, the more positive the number, the easier it is for the receptor–ligand complex to dissociate. The table below shows that all the compound–protein interactions evaluated in this study were negative and that the catalase protein displayed the highest affinities with the seven phenolic compounds.

**Table 9:** Average results of the dissociation constant between the main phenolic compounds and the proteins of the apoptosis pathway.

Compound/(ligand)	*Trans*-resveratrol	Gallic acid	Ferulic acid	Caffeic acid	Quercetin	Catechin	Malvidin
Protein/(receptor)							
*HSP27*	−7.2	−6.4	−7.2	−7.2	−8.5	−8.1	−7.5
*Standard deviation*	0.41	0.45	0.25	0.10	0.28	0.25	0.40
*Bcl-xL*	−9.9	−6.3	−7.0	−7.0	−9.4	−9.1	−9.3
*Standard deviation*	0.15	0.23	0.05	0.20	0.15	0.15	0.15
*Catalase*	−8.9	−7.3	−7.1	−7.7	−10.8	−10.2	−9.6
*Standard deviation*	0.25	0.10	0.11	0.15	0.10	0.32	0.30
*TRAIL R1/DR4*	−7.0	−7.0	−6.3	−6.0	−8.1	−7.6	−6.9
*Standard deviation*	0.10	0.10	0.15	0.10	0.35	0.20	0.10
*Smac/DIABLO*	−5.6	−4.2	−4.9	−4.9	−6.2	−6.5	−8.2
*Standard deviation*	0.15	0.25	0.05	0.1	0.15	0.20	0.05
*Survivin*	−7.3	−5.3	−6.2	−6.4	−7.1	−7.1	−7.3
*Standard deviation*	0.10	0.20	0.10	0.05	0.10	0.15	0.25

**Fig. 11:**
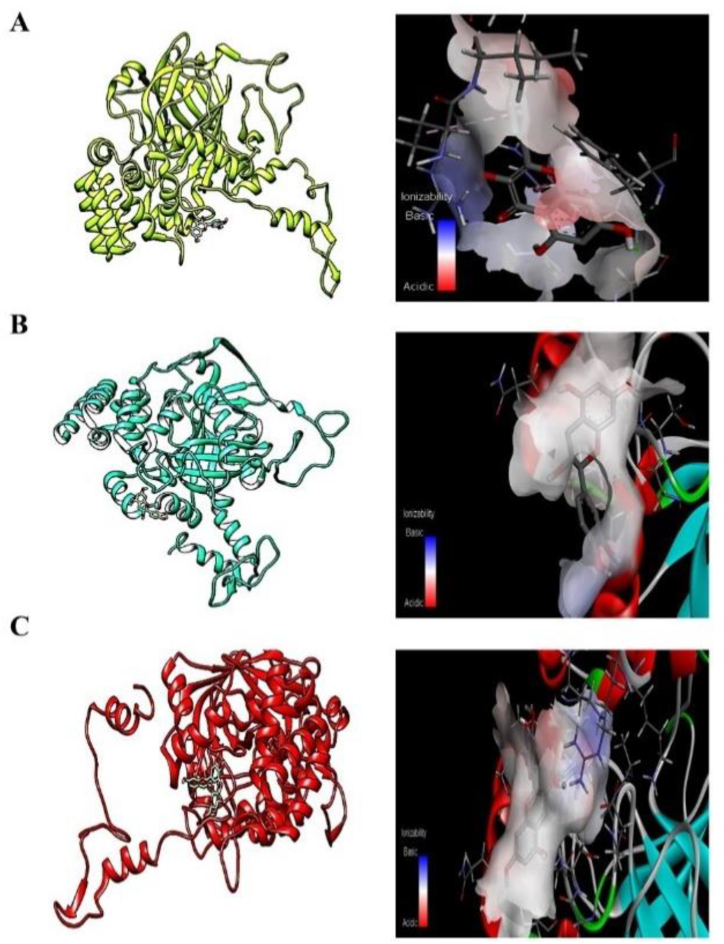
Interactions of catalase with (A) quercetin, (B) catechin, and (C) malvidin. Three simulations were performed for each interaction and the average is reported in Table 5. The left side shows the part of the protein that interacts with the ligand. The right side shows the pH of the interaction site, where red indicates acidic pH; blue, basic pH; and white, neutral pH.

Fig. 11 shows the chemical configuration of the catalase protein and its binding site with quercetin in Fig. 11A, catechin in Fig. 11B, and malvidin in Fig. 11C. After the simulation, these were the ligands with the lowest dissociation constant. In the models presented below, the pH of the binding site for the three compounds is constant and neutral.

**Fig. 12:**
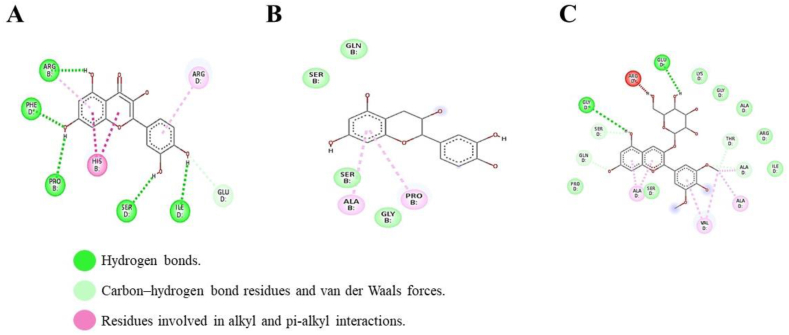
Type of bond formed between the amino acids of the catalase protein and the amino acids present in three phenolic compounds: (A) quercetin, (B) catechin, and (C) malvidin.

Fig. 12 presents the results of the interactions with higher affinity with catalase for quercetin in Fig. 12A, catechin in Fig. 12B, and malvidin in Fig. 12C. Likewise, the characteristics of the receptor-ligand binding sites are detailed, such as hydrogen bonds, carbon-hydrogen bond residues, and van der Waals forces.

The results obtained from the simulations conducted in this study show that Isabella grape extract produces a lower dissociation constant between the catalase protein and the phenolic compounds (i.e., quercetin, catechin, and malvidin). Phenolic compounds can activate or deactivate the expression of certain proteins, which can, in turn, activate or deactivate signaling cascades in the apoptosis pathway [107]. In addition, phenolic compounds have been described as molecules with antioxidant capacity. Catalase is an important antioxidant protein that controls the level of oxidative stress in the human body [108,109]. This protein is a potential target for the treatment of different types of cancer, which is why drug designers try to protect its catalytic activity in diverse physiological conditions [108]. The drug–protein interaction of catalase has received special attention because it has been established that the formation of these complexes influences cell damage, death, or apoptosis. Previous studies have demonstrated that this enzyme plays an important role in cancer and the body's response to drugs used against this disease [[110], [111], [112]]. Consequently, detecting and determining catalase activity is useful in estimating the influence of multiple anticancer drugs [107]. *In silico* molecular dynamics studies should be continued to better characterize the interactions and further characterization of the grape extract phenolic compounds should be conducted to evidence its potential modulatory activity on apoptotic pathways.

In conclusion, this study optimized an extract obtained from *V. labrusca* that, due to its polyphenol content and antioxidant capacity, shows a potential chemopreventive effect on breast cancer. The evidence indicates that said extract induced a decrease in the cell viability of the MCF-7 line, which was associated with mitochondrial membrane depolarization, ROS increase, and chromatin condensation. Additionally, an *in silico* assay was conducted to observe molecular interactions between the phenolic compounds found in Isabella grape and proteins associated with apoptotic pathways. Further studies should be carried out to deepen our understanding of the biological effect of this extract on this cell line and other cancer models.

## Author contribution statement

M. Daniela Vélez: María A. Llano-Ramirez: Carolina Ramón: Performed the experiments; Analyzed and interpreted the data; Wrote the paper.

Jessica Rojas: Analyzed and interpreted the data; Wrote the paper.

Carolina Bedoya: Performed the experiments; Analyzed and interpreted the data.

Sandra Arango-Varela: Gloria A Santa-Gonzalez: Maritza Gil: Conceived and designed the experiments; Performed the experiments; Analyzed and interpreted the data; Contributed reagents, materials, analysis tools or data; Wrote the paper.

## Data availability statement

Data will be made available on request.

## Declaration of competing interest

The authors declare that they have no known competing financial interests or personal relationships that could have appeared to influence the work reported in this paper

## Abbreviations

AAPH: 2,2′-azobis (2 -amidinopropane) dihydrochloride
AH: Antioxidant concentration
ANOVA: Analysis of variance
AOAC: Association of Official Agricultural Chemists
AUC: Area under the curve
CAS (number): Chemical Abstracts Service (number)
DF: Dilution factor
DiOC_6_: 3,3′-dihexyloxacarbocyanine iodide
DMEM: Dulbecco's Modified Eagle's Medium
DNA: Deoxyribonucleic acid
FBS: Fetal bovine serum
GAE: Milligrams of gallic acid equivalents
IAP: Inhibitor of apoptosis proteins
IC_50_: Concentration of a drug that gives half-maximal response
MAC: Monomeric anthocyanin content
MFI: Mean fluorescence intensity
MTT: (3-(4,5-Dimethylthiazol-2-yl)-2,5-Diphenyltetrazolium Bromide)
MW: Molecular weight
ORAC: Oxygen radical absorbance capacity
PBS: Phosphate-buffered saline
PDB: Protein Data Bank
PRM: Polynomial Regression Model
PI: Propidium iodide
SD: Standard deviation
ROS: Reactive oxygen species
SX: Selectivity index
TPC: Total polyphenol content
USAE: Ultrasound-assisted extraction
