# Supplementary material for: Antioxidant capacity and cytotoxic effect of an optimized extract of isabella grape (Vitis labrusca) on breast cancer cells
Source: Heliyon. 2023 May 24;9(6):e16540. doi: 10.1016/j.heliyon.2023.e16540 (PMC10227348; doi:10.1016/j.heliyon.2023.e16540)
Supplement: Multimedia component 1 [file mmc1.docx]

**Table 1S.** P-value of the pairwise comparison analysis between the means of the total polyphenol content values obtained from the evaluation of the different solvent systems studied, determined by the Tukey HSD test.

| **Solvents Systems** | **P-value** |
| --- | --- |
| Water: Ethanol (1:0) | 0.0000000 |
| Water:Ethanol (0:1) | 0.5284841 |
| Water:Ethanol (0.5:0.5) | 0.0000000 |

P-values<0.05 indicate significant differences
