# Supplementary material for: Antioxidant capacity and cytotoxic effect of an optimized extract of isabella grape (Vitis labrusca) on breast cancer cells
Source: Heliyon. 2023 May 24;9(6):e16540. doi: 10.1016/j.heliyon.2023.e16540 (PMC10227348; doi:10.1016/j.heliyon.2023.e16540)
Supplement: Multimedia component 2 [file mmc2.docx]

**Table 2S.** P-value of the pairwise comparison analysis between the means of the total polyphenol content values obtained at the different extraction times evaluated (15, 30, 45, 60, 75, 90, 105, 120, 135 min) at two frequencies tested (33 kHz and 40 kHz), determined by the Tukey HSD test.

| **Comparisons**  **(Time - min)** | **P-value** | |
| --- | --- | --- |
|  | **33 kHz** | **40 kHz** |
| 30-15 | 0,0209632 | 0,0000007 |
| 45-15 | 0,0000334 | 0,0143441 |
| 60-15 | 0,0000002 | 0,0000057 |
| 75-15 | 0,0000000 | 0,0000005 |
| 90-15 | 0,0000000 | 0,0000002 |
| 105-15 | 0,0000000 | 0,0000151 |
| 120-15 | 0,0105821 | 0,9890047 |
| 135-15 | 0,0017219 | 0,0000392 |
| 45-30 | 0,2661990 | 0,0143441 |
| 60-30 | 0,0030266 | 0,9943189 |
| 75-30 | 0,0000004 | 1,0000000 |
| 90-30 | 0,0000753 | 0,9998384 |
| 105-30 | 0,0000060 | 0,9433240 |
| 120-30 | 0,9999983 | 0,0000072 |
| 135-30 | 0,0000001 | 0,0000000 |
| 60-45 | 0,5558762 | 0,0967510 |
| 75-45 | 0,0002044 | 0,0095660 |
| 90-45 | 0,0435911 | 0,0040349 |
| 105-45 | 0,0039300 | 0,2045344 |
| 120-45 | 0,4108661 | 0,1161584 |
| 135-45 | 0,0000000 | 0,0000000 |
| 75-60 | 0,0319630 | 0,9820349 |
| 90-60 | 0,8781948 | 0,9064127 |
| 105-60 | 0,3144774 | 0,9999805 |
| 120-60 | 0,0061829 | 0,0000611 |
| 135-60 | 0,0000000 | 0,0000000 |
| 90-75 | 0,4716002 | 0,9999919 |
| 105-75 | 0,9615968 | 0,8914518 |
| 120-75 | 0,0000007 | 0,0000047 |
| 135-75 | 0,0000000 | 0,0000000 |
| 105-90 | 0,9833917 | 0,7204706 |
| 120-90 | 0,0001565 | 0,0000020 |
| 135-90 | 0,0000000 | 0,0000000 |
| 120-105 | 0,0000122 | 0,0001662 |
| 135-105 | 0,0000000 | 0,0000000 |
| 135-120 | 0,0000001 | 0,0000037 |

P-values<0.05 indicate significant differences
